# Thyroid Function, Inflammatory Response, and Glucocorticoids in COVID-19

**DOI:** 10.3389/fendo.2022.939842

**Published:** 2022-07-29

**Authors:** Renata Świątkowska-Stodulska, Agata Berlińska, Ewelina Puchalska-Reglińska

**Affiliations:** ^1^ Department of Endocrinology and Internal Medicine, Faculty of Medicine, Medical University of Gdańsk, Gdańsk, Poland; ^2^ Dialysis Unit, 7 Navy Hospital, Gdańsk, Poland

**Keywords:** thyroid, COVID-19, coronavirus disease 2019, SARS CoV-2, free triiodothyronine, interleukin-6, glucocorticoids, TSH

## Abstract

The ongoing COVID-19 pandemic calls for extensive research on various medical topics. Since the beginning of the pandemic, multiple studies investigated the impact of SARS CoV-2 on thyroid function. However, crucial data, such as trend progression over time or influence of commonly used drugs, might still be missing. We checked the thyroid function in 174 patients with PCR-confirmed COVID-19. Our research covered three separate time points of hospitalization (days 1, 4, and 10). We did not exclude patients treated with glucocorticoids but, instead, compared them with patients not treated with steroids. We correlated the results of thyroid function tests with markers of systemic inflammation. We checked if abnormal thyroid function can predict unfavorable outcomes defined as combined primary endpoint and/or secondary endpoints; the combined primary endpoint was the occurrence of death, mechanical ventilation, non-invasive ventilation, vasopressor infusion, or prolonged hospital stay, and the secondary endpoint was any of the listed events. In general, 80.46% of evaluated patients displayed abnormalities in thyroid function tests over at least one time point throughout the observation. We noticed a high prevalence of features typical for thyroid dysfunction in non-thyroidal illness (NTI). Free triiodothyronine (fT3) concentration was significantly lower in the group requiring glucocorticoids. Patients displaying abnormal thyroid function were statistically more likely to meet the predefined combined primary endpoint. We found that fT3 measured at admission could be perceived as an independent predictor of endpoint completion for all analyzed groups. Thyroid involvement is common in COVID-19. Our study supports the idea of thyroid function abnormalities being important clinical tools and allowing early recognition of possible detrimental outcomes of the disease.

## Introduction

Over the past two years of the COVID-19 pandemic, the thyroid function in the context of COVID-19 quickly gained wide attention of researchers. As early as in the mid-2020, first studies on the topic were published. Chen and colleagues, the authors of one of the first articles, noticed that thyrotropin (TSH) and total triiodothyronine (T3) in COVID-19 patients were significantly lower than in their counterparts who either suffered from non-COVID-19 pneumonia or were healthy (1). Subsequent studies confirmed the increased occurrence of features typical for thyroid dysfunction in nonthyroidal illness (NTI); however, the abnormalities were rather mild and rarely required specific treatment (2). A large prospective study conducted by Beltrão et al. concluded that early evaluation of simple biomarkers such as free triiodothyronine (fT3) and reverse triiodothyronine (rT3) in patients with moderate to severe COVID-19 can be helpful in assessment of the overall prognosis (3). A systematic review of 1237 patients enrolled into 7 studies performed by Giovanella et al. proved thyroid dysfunction in COVID-19 to be common, ranging between 13 and 64% of cases, and once again called attention to the positive correlation between the disease severity and abnormal thyroid function (4). Extensive reviews of COVID-19-related thyroid pathologies performed by Murugan and Alzahrani further highlighted the depth and prevalence of observed anomalies, calling for a careful evaluation of potentially affected individuals, with special regards for, amongst others, NTI, autoimmune thyroiditis, and subacute thyroiditis (5, 6). Data pointing out high incidence of new onset of hyperthyroidism (up to 20% of examined cases) in COVID-19 was presented (7). Currently, it is believed that observed thyroid pathologies arise not only from the well-known phenomena such as the thyroid dysfunction in NTI, but also from the direct action of SARS CoV-2 on thyrocytes and thyrotrope pituitary cells (6, 8–13).

Most of the available studies deliver the results of thyroid function tests assessed over a single time point and often correlate them with markers of inflammation (2, 14, 15). In our project, the tests were carried out over three separate time points throughout the hospital stay. Also, we aimed at providing information about possible relationships between the thyroid function and the occurrence of unfavorable endpoints (prolonged hospital stay, non-invasive or mechanical ventilation, infusion of vasoactive amines, and death) and about potential links between the concentration of hypothalamus-pituitary-thyroid (HPT) hormones and inflammatory markers.

## Materials and Methods

We designed a multiple cross-sectional study which recruited 180 adult patients (≥18 years old) with PCR-confirmed COVID-19 (nasopharyngeal swabs). All patients required hospital stay at the 7 Navy Hospital in Gdańsk, Poland, which served as our recruitment center between the 14 February 2021 and the 1 December 2021. 6 patients were excluded from the final analysis due to essential data missing which left us with 174 patients for the final analysis.

We gathered information about the patients’ metrical and anthropometric data, as well as previous medical history. Prospectively, we evaluated oxygen supplementation, glucocorticoid use, vital parameters, the occurrence of unfavorable events (death, mechanical and/or non-invasive ventilation, vasopressor use), length of hospitalization, and laboratory tests (TSH, total thyroxine [T4], free thyroxine [fT4], fT3, rT3, anti-thyroperoxidase antibodies [anti-TPO abs], anti-thyroglobulin antibodies [anti-TG abs], leukocyte count [LEU], neutrocyte count [NEU], lymphocyte count [LYMPH], interleukin 6 [IL-6], C-reactive protein [CRP]).

Laboratory assays were performed using venous blood collected between 6 AM and 8 AM. There were three separate dates for blood collection, later referred to as the time points: day 1, 4, and 10 of the hospital stay. The blood collection could be moved up to 24 hours, however, we highly encouraged timely procedures. On all three dates, we assessed TSH, T4, fT4, fT3, rT3, CRP, IL-6, LEU, NEU, and LYMPH. In addition, on day 1 we checked anti-TG abs and anti-TPO abs.

Laboratory tests were performed by commercial laboratories. Due to technical abilities at the time of recruitment, rT3 measurement was performed in two separate laboratories. Information on laboratory methods is available in **Table 1**.

**Table 1:** Detailed technical information on used laboratory assays.

Parameter		Reference norm with units	Laboratory	Analytical method	Analyzer
1.	TSH	0,27-4,2 μIU/ml	Diagnostyka Laboratoria, Gdańsk, Poland	Electrochemiluminescence assay	Cobas 8000, Roche
2.	fT4	12-22 pmol/l	Diagnostyka Laboratoria, Gdańsk, Poland	Electrochemiluminescence assay	Cobas 8000, Roche
3.	fT3	3,1-6,8 pmol/l	Diagnostyka Laboratoria, Gdańsk, Poland	Electrochemiluminescence assay	Cobas 6000, Roche
4.	rT3	i. <0,95 ng/ml ii. 0,1-0,35 μg/l	i. Central Clinical Laboratory, University Clinical Centre, Gdańsk, Poland ii. Cerba International, Barcelona, Spain	i. Chemiluminescence immunoassay ii. Chemiluminescence immunoassay	i. Maglumi 800, Snibe ii. Maglumi 800, Snibe
5.	T4	5,13-14,10 µg/dl	Diagnostyka Laboratoria, Gdańsk, Poland	Electrochemiluminescence assay	Cobas, Roche
6.	anti-TPO abs	<9 IU/ml	Diagnostyka Laboratoria, Gdańsk, Poland	Electrochemiluminescence assay	Cobas 8000, Roche
7.	anti-TG abs	<10 IU/ml	Diagnostyka Laboratoria, Gdańsk, Poland	Electrochemiluminescence assay	Cobas 8000, Roche
8.	CRP	0-5 mg/l	Diagnostyka Laboratoria, Gdańsk, Poland	Immunoturbidimetric assay	Cobas 6000, Roche
9.	IL-6	<5.9 pg/ml	Central Clinical Laboratory, University Clinical Centre, Gdańsk, Poland	Chemiluminescence immunoassay	Immulite XP, Siemens
10.	LEU	females: 3.98-10.04x10^3^/µl males: 4.23-9.07x10^3^/µl	Diagnostyka Laboratoria, Gdańsk, Poland	*Fluorescence flow cytometry*	XT-4000i, Sysmex
11.	NEU	2-7x10^3^/µl	Diagnostyka Laboratoria, Gdańsk, Poland	*Fluorescence flow cytometry*	XT-4000i, Sysmex
12.	LYMPH	1-3x10^3^/µl	Diagnostyka Laboratoria, Gdańsk, Poland	*Fluorescence flow cytometry*	XT-4000i, Sysmex

Some of the recruited patients required glucocorticoids (GCs) for COVID-19 or chronic diseases. Exogenous GC use modifies the endocrine function, therefore, for the sake of proper data analysis, we separated the patients who received GCs (the glucocorticoid group – GCG; N = 93) from those who did not (the no-glucocorticoid group – NGCG; N = 81). The GCs were either dexamethasone or methylprednisolone. Patients were labeled as the GCG if they received parenteral and/or oral GCs at least once during their stay and GCs were administered at least one day before the blood was collected.

We conducted the study in accordance with the Declaration of Helsinki. Our project was approved by the Independent Bioethics Committee for Scientific Research at the Medical University of Gdańsk (permissions NKBBN/373/2020, NKBBN/373-96/2021, NKBBN/373-184/2021). Written consent was obtained by the recruiters. As some patients arrived in bad general condition due to COVID-19 and/or concomitant diseases, they were unable to give written consent. In such cases, recruiters made a clear remark on the consent form. All recruited patients maintained their consent to participation. Inclusion criteria consisted of PCR-confirmed COVID-19, age of at least 18 years, and consent to participation. There were no exclusion criteria other than dissent to participation. The study was registered at ClinicalTrials.gov (NCT05070091).

## Statistical Analysis

The Centre of Biostatistics and Bioinformatics Analysis, the Medical University of Gdańsk in Gdańsk, Poland performed the analysis. The analysis was performed using the R environment, with additional use of MS Excel. The threshold of significance was defined as p<0.05.

Quantitative data was evaluated using the Shapiro-Wilk W test and, based on the distribution pattern, was presented as either arithmetic mean with standard deviation (normal distribution) or as median with interquartile range (distribution deviating from normal). To describe qualitative data, absolute numbers and percentages were used.

The Spearman’s test for rank correlation was applied to correlate the available variables, with the Spearman’s r_SP_ correlation coefficient was used to check the strength of correlations. By applying the coefficient of determination R^2^, the proportion of variation explained by correlation was calculated. The groups were compared using the Mann-Whitney U test. Associations between variables were checked using the Cramer’s phi, the Yule’s Q, and the Kendall’s tau tests.

The changes in hormone levels over time were determined by the repeated measures ANOVA (rmAOV). The results were later validated using the Mauchly’s sphericity test and, sometimes, adjusted using the Greenhouse-Geisser correction. The Student’s t test was introduced for *post-hoc* multiple comparisons, with the FDR correction by Benjamini and Hochberg used whenever necessary.

Logistic regression and ROC curve assessment were used to check if baseline fT3 can be seen as an early predictor of unfavorable endpoints. The relationship between thyroid dysfunction and endpoints was validated by calculating the odds ratio (OR) with a 95% confidence interval. OR was measured using the Fisher’s exact test and logistic regression.

Analyses covering rT3 were carried out separately depending on the laboratory site.

## Results

The final analysis included 174 patients: 93 from the NGCG and 81 from the GCG. In the NGCG, 12 patients had a history of hypothyroidism and 11 received levothyroxine (LT4); one patient required anti-thyroid treatment due to hyperthyroidism. In the GCG, 6 patients had a history of hypothyroidism and 4 required LT4; one patient used thyrostatics for hyperthyroidism. All patients with a history of thyroid dysfunction were clinically euthyroid at the time of recruitment. 7 patients (4.0%) required GCs for chronic conditions other than COVID-19 (4 – dexamethasone, and 3 – methylprednisolone); in this group, GCs were maintained as usual.

The mean age of recruited patients was 66.77 ±15.06 years, age range 18-94 years. 84 out of 174 patients (48.3%) were women. Half of the patients (87 out of 174 – 50.0%) required hospital stay lasting 10 days or longer. 102 (58.62%) patients suffered from arterial hypertension, 53 (30.46%) – type 2 diabetes, 17 (9.77%) – heart failure, 23 (13.22%) – atrial fibrillation, 30 (17.24%) – coronary artery disease, 18 (10.34%) – asthma and/or chronic obstructive pulmonary disease, 26 (14.94%) – chronic kidney disease, 17 (9.77%) required hemodialysis, 11 (6.32%) had a history of stroke, 3 (1.72%) – pulmonary embolism, and 14 (8.05%) – active neoplasia. BMI of 139 patients was analyzed – mean BMI equaled 27.57 ± 5.25 kg/m^2^, with 53 (38.1%) individuals being overweight, and 39 (28.1%) – obese. Supplemental oxygen was introduced in 100 out of 173 subjects (57.8%). Over the period of observation, 30 patients (17.2%) died, 12 (6.9%) needed NIV/CPAP and/or HFNO, 3 (1.7%) – ventilator, and 6 (3.5%) – vasopressor infusion.

We assessed the thyroid function of patients in three predefined time points. The normal range was based on the recommendations provided by the laboratory kits’ producers (**Table 1**). The parameters we considered were TSH, T4, fT4, fT3, and rT3, with an addition of anti-TG and anti-TPO abs. The results were labeled abnormal if any of the parameters deviated from the laboratory norms. In some patients, the clinical and/or laboratory data were incomplete which is reflected by the number of studied cases (N).

140 out of 174 patients (80.46%) displayed abnormal thyroid function in at least one time point throughout the observation. In time point #1, abnormal thyroid function was present in 33 out of 56 (58.9%) patients from the GCG and in 67 out of 118 (56.8%) patients from the NGCG. In time point #2, thyroid dysfunction was present in 51 out of 74 (68.9%) patients from the GCG and in 48 out 83 (57.8%) patients from the NGCG. In time point #3, thyroid function was abnormal in 49 out of 63 (77.8%) GCG patients and in 33 out of 46 (71.7%) NGCG patients (**Table 2**).

**Table 2:** Pattern of thyroid function abnormalities throughout the observation time.

Type of abnormalities	NGCG			GCG		
	Time point #1 (number/percentage of cases)	Time point #2 (number/percentage of cases)	Time point #3 (number/percentage of cases)	Time point #1 (number/percentage of cases)	Time point #2 (number/percentage of cases)	Time point #3 (number/percentage of cases)
Total	67/56.78%	48/57.83%	33/71.74%	33/58.93%	51/68.92%	49/77.78%
Thyroid dysfunction in non-thyroidal illness	51/43.22%	32/38.55%	20/43.48%	29/51.78%	40/54.05%	42/66.67%
Unspecified/mixed	16/13.56%	16/19.28%	13/28.26%	4/7.14%	11/14.86%	7/11.11%

**Figure 1:**
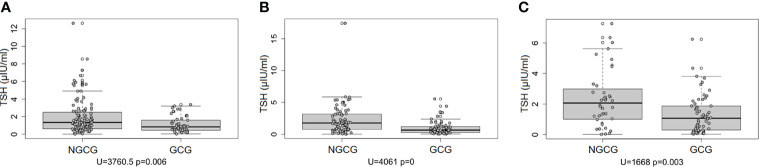
Concentration of TSH over three consecutive time points among patients not treated with glucocorticoids (NGCG) and treated with glucocorticoids (GCG). **(A)** time point #1; **(B)** time point #2; **(C)** time point #3.

**Figure 2:**
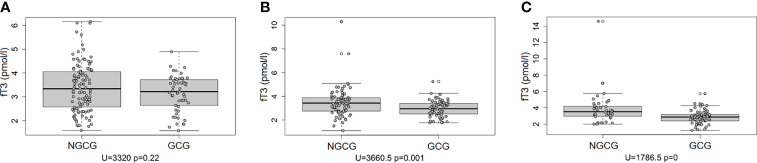
Concentration of fT3 over three consecutive time points among patients not treated with glucocorticoids (NGCG) and treated with glucocorticoids (GCG). **(A)** time point #1; **(B)** time point #2; **(C)** time point #3.

Next, we focused on the connection between the thyroid function and the GC use: we found statistically significant differences between the GCG and the NGCG. TSH concentration was lower in the GCG as compared with the NGCG in all three time points (time point #1: p<0.01; time point #2: p<0.0001; time point #3: p<0.05) – **Figure 1**. Levels of fT3 were significantly lower in the GCG than in the NGCG in time points #2 and #3 (time point #2: p=0.001, time point #3: p<0.0001) – **Figure 2**. We observed a statistically relevant decrease in T4 concentration in the GCG as compared with the NGCG only in time point #3 (p<0.05). There were no relevant differences in fT4 and rT3 levels between the GCG and the NGCG in any of the time points (**Tables 3**–**5**).

**Table 3:** Chosen clinical and laboratory parameters – time point #1.

Characteristics	Time point #1											
	NGCG						GCG					
	N	Mean ± SD	LQ	Medium	UQ	Range	N	Mean ± SD	LQ	Medium	UQ	Range
Age (years)	118	67.229 ± 15.134	58.000	69.000	79.000	18.00-94.00	56	65.804 ± 14.984	56.750	68.000	75.750	31.00-90.00
BMI (kg/m^2^)	95	26.815 ± 5.146	23.620	25.760	29.530	17.33-40.89	44	29.213 ± 5.156	25.620	27.895	31.328	19.53-44.08
Oxygen demand (l/min.)	117	2.517 ± 5.237	0.000	0.000	4.000	0.00-30.00	56	10.562 ± 15.958	3.000	6.000	10.000	0.00-75.00
Hospital stay (days)	118	12.890 ± 9.135	7.000	10.000	18.000	1.00-50.00	56	10.429 ± 6.126	6.000	9.000	14.250	1.00-30.00
TSH (μIU/ml)	112	1.916 ± 1.9699	0.619	1.330	2.428	0.005-12.62	53	1.117 ± 0.900	0.444	0.827	1.590	0.025-3.37
fT3 (pmol/l)	112	3.389 ± 1.017	2.582	3.340	4.043	1.590-6.15	53	3.145 ± 0.756	2.640	3.220	3.720	1.580-4.89
fT4 (pmol/l)	112	15.357 ± 3.352	13.180	14.620	16.900	9.660-27.07	53	15.132 ± 3.384	13.440	14.700	17.540	4.880-23.05
T4 (μg/dl)	110	7.570 ± 1.791	6.385	7.520	8.595	4.240-13.00	52	7.575 ± 2.321	5.885	7.185	9.045	2.380-14.5-
rT3 – laboratory #1 (μg/l = ng/ml)	37	0.219 ± 0.052	0.190	0.210	0.240	0.130-0.35	12	0.202 ± 0.041	0.170	0.190	0.220	0.160-0.29
rT3 – laboratory #2 (ng/ml = μg/l)	72	0.652 ± 0.239	0.478	0.605	0.755	0.270-1.35	40	0.605 ± 0.315	0.465	0.555	0.685	0.080-1.90
anti-TG abs (IU/ml)	112	75.477 ± 395.412	3.200	3.200	11.800	3.200-4000.00	53	12.136 ± 24.652	3.200	3.200	11.600	3.200-141.00
anti-TPO abs (IU/ml)	112	21.841 ± 75.617	3.000	3.000	12.425	3.000-589.00	52	17.151 ± 51.859	3.000	3.000	12.000	3.000-346.00
IL-6 (pg/ml)	111	35.888 ± 47.938	9.390	19.400	41.650	1.410-267.00	53	233.631 ± 1368.543	7.210	20.000	59.100	1.41-10,000.00
CRP (mg/l)	112	61.400 ± 67.871	9.875	39.700	84.775	0.700-320.10	53	103.50 ± 100.612	21.700	78.100	139.900	1.00-427.70
LEU (^10^3^/µl)	112	6.786 ± 3.578	4.375	5.845	8.152	1.790-26.16	53	6.706 ± 3.823	3.920	5.590	8.280	1.90-21.22
NEU (^10^3^/µl)	112	4.640 ± 3.241	2.570	3.560	5.610	1.220-21.36	53	5.29 ± 3.670	3.120	4.390	6.520	1.08-20.24
LYMPH (^10^3^/µl)	112	1.332 ± 0.652	0.858	1.260	1.755	0.260-3.56	53	0.898 ± 0.499	0.650	0.800	1.030	0.17-2.78

**Table 4:** Chosen clinical and laboratory parameters – time point #2.

Characteristics	Time point #2											
	NGCG						GCG					
	N	Mean ± SD	LQ	Medium	UQ	Range	N	Mean ± SD	LQ	Medium	UQ	Range
Age (years)	83	68.157 ± 14.374	60.000	70.000	78.500	18.00-94.00	74	65.459 ± 15.664	56.000	67.000	78.000	31.00-94.00
BMI (kg/m^2^)	67	26.533 ± 4.877	23.775	25.950	28.360	17.40-40.82	58	28.752 ± 5.501	24.828	27.815	31.250	17.33-44.08
Oxygen demand (l/min.)	82	2.293 ± 6.787	0.000	0.000	0.000	0.00-40.00	73	8.493 ± 11.168	0.000	5.000	11.000	0.00-60.00
Hospital stay (days)	83	13.458 ± 9.793	7.000	10.000	18.500	2.00-50.00	74	12.730 ± 5.836	8.000	12.000	16.000	4.00-30.00
TSH (μIU/ml)	80	2.252 ± 2.365	0.787	1.420	2.770	0.032-17.42	70	0.964± 1.030	0.289	0.656	1.202	0.051-5.55
fT3 (pmol/l)	79	3.506 ± 1.235	2.755	3.430	3.895	1.100-10.29	70	2.964 ± 0.683	2.520	2.955	3.400	1.780-5.24
fT4 (pmol/l)	80	15.709 ± 3.480	13.453	15.380	17.355	9.870-31.69	70	16.117± 3.499	13.518	15.830	18.677	7.050-24.43
T4 (μg/dl)	78	7.828 ± 1.683	6.628	7.745	8.900	4.690-13.60	70	7.813 ± 2.194	6.418	7.730	8.968	3.370-16.00
rT3 – laboratory #1 (μg/l = ng/ml)	18	0.198 ± 0.068	0.150	0.180	0.232	0.120-0.35	16	0.213 ± 0.057	0.180	0.205	0.222	0.140-0.38
rT3 – laboratory #2 (ng/ml = μg/l)	56	0.726 ± 0.319	0.528	0.635	0.843	0.330-2.24	50	0.645± 0.230	0.472	0.620	0.775	0.270-1.34
IL-6 (pg/ml)	78	41.296 ± 62.231	8.975	21.000	48.275	1.41-399.00	70	39.765 ± 167.153	3.990	7.920	18.100	1.41-1374.00
CRP (mg/l)	79	52.347 ± 60.984	9.150	36.300	66.000	0.70-281.80	70	40.333 ± 41.274	13.875	24.750	57.425	0.80-171.30
LEU (^10^3^/µl)	80	6.576 ± 3.230	4.355	5.615	8.035	0.56-18.25	69	7.460 ± 2.861	5.200	7.500	10.090	2.39-13.32
NEU (^10^3^/µl)	80	4.392 ± 2.997	2.375	3.250	5.862	0.25-14.31	69	5.658 ± 2.522	3.450	5.880	7.270	1.33-11.66
LYMPH (^10^3^/µl)	80	1.331 ± 0.676	0.880	1.200	1.765	0.12-3.92	69	1.040 ± 0.585	0.670	0.920	1.230	0.35-3.42

**Table 5:** Chosen clinical and laboratory parameters – time point #3.

Characteristics	Time point #3											
	NGCG						GCG					
	N	Mean ± SD	LQ	Medium	UQ	Range	N	Mean ± SD	LQ	Medium	UQ	Range
Age (years)	46	68.435 ± 14.842	61.500	70.000	75.000	18.00-94.00	63	68.365 ± 14.544	61.500	69.000	80.000	31.00-94.00
BMI (kg/m^2^)	38	26.054 ± 4.267	22.868	25.735	28.263	19.10-38.30	49	29.302 ± 5.980	24.690	27.940	32.030	17.33-44.08
Oxygen demand (l/min.)	41	1.976 ± 5.303	0.000	0.000	0.000	0.00-30.00	61	4.984 ± 9.856	0.000	0.000	6.000	0.00 -52.00
Hospital stay (days)	46	15.565 ± 8.400	9.000	13.500	20.000	4.00-41.00	63	15.952 ± 8.261	10.000	14.000	19.000	6.00- 50.00
TSH (μIU/ml)	40	2.344 ± 1.886	1.017	2.060	2.875	0.006-7.26	62	1.311 ± 1.239	0.314	1.065	1.850	0.024-6.24
fT3 (pmol/l)	40	3.863 ± 2.055	2.982	3.515	4.162	2.000-14.59	62	2.845 ± 0.833	2.405	2.865	3.165	1.230-5.73
fT4 (pmol/l)	40	16.401 ± 5.558	13.662	15.570	17.320	9.290-44.12	62	16.319 ± 3.989	13.625	16.595	19.125	6.710-25.38
T4 (μg/dl)	40	8.338 ± 2.462	7.312	7.880	9.032	4.200-17.40	61	7.158 ± 2.056	5.730	7.250	8.260	2.910-12.62
rT3 – laboratory #1 (μg/l = ng/ml)	11	0.184 ± 0.043	0.155	0.170	0.200	0.140-0.26	14	0.197 ± 0.052	0.162	0.190	0.230	0.120-0.31
rT3 – laboratory #2 (ng/ml = μg/l)	26	0.919 ± 0.547	0.650	0.815	1.070	0.320-3.27	45	0.734 ± 0.835	0.460	0.560	0.720	0.110-5.88
IL-6 (pg/ml)	40	47.343 ± 115.409	8.705	16.900	29.275	1.41-704.00	61	140.251 ± 917.906	3.530	8.130	22.200	1.41-7182.00
CRP (mg/l)	40	47.150 ± 54.222	8.550	32.800	58.500	0.80-280.30	62	37.098 ± 67.678	3.450	7.900	30.700	0.80-342.60
LEU (^10^3^/µl)	40	6.558 ± 2.678	4.920	5.770	7.195	1.92-16.96	62	10.039 ± 5.770	6.478	9.230	11.925	3.09-39.01
NEU (^10^3^/µl)	40	4.224 ± 2.734	2.638	3.385	4.970	0.68-15.84	62	7.702 ± 5.548	4.395	6.585	9.047	1.36-35.97
LYMPH (^10^3^/µl)	40	1.352 ± 0.621	0.865	1.290	1.768	0.43-3.19	62	1.287 ± 0.756	0.747	1.185	1.585	0.28-4.46

As the next step of our analysis, we checked the relationship between the thyroid function and the occurrence of unfavorable clinical outcomes defined as the endpoints. The combined primary endpoint was a composite of hospitalization ≥10 days, mechanical ventilation, non- invasive ventilation (CPAP/NIV) and/or high-flow nasal oxygen device (HFNO), vasopressor use, death. The secondary endpoints were defined as any of the conditions listed above. 95 out of 140 (67.86%) patients with abnormal thyroid function and 12 out of 34 (35.29%) patients with normal thyroid function met the combined primary endpoint.

Patients displaying abnormal thyroid function were statistically more likely to meet the combined primary endpoint (OR=3.9, p=0.0001); among the secondary endpoints, statistical significance was found for prolonged hospitalization (OR=5.1, p<0.0005). When the confounders, such as age, sex, and BMI, were considered, the ORs were even higher (OR=5.3, p=0.001 and OR=6.6, p=0.001, consecutively). No significant relationship between the thyroid function and secondary endpoints other than prolonged hospital stay was found.

We checked if the baseline fT3 concentration can predict the endpoints. The analysis was performed for the entire studied population, as well as for the GCG and the NGCG separately. We found that fT3 measured at admission might predict the occurrence of endpoints in all analyzed groups (the general population: regression coefficient -1.067, p=0.0000; the GCG: regression coefficient -1.286, p=0.0010; the NGCG: regression coefficient -0.936, p=0.0051). Statistical significance was found regardless of the confounders (age, sex, BMI). Negative values of the regression coefficients suggested that the higher the fT3 concentration, the lower the risk of unfavorable endpoints.

Next, possible correlations between thyroid function and markers of generalized inflammation were assessed. For the first time point, significant negative correlations were found between TSH and CRP (r=-0.169, p=0.030), fT3 and CRP (r=-0.398, p=0.000), fT3 and IL-6 (r=-0.262, p=0.001), fT3 and LEU (r=-0.237, p=0.002), T4 and IL-6 (r=-0.182, p=0.022), T4 and LEU (r=-0.189, p=0.016), and rT3 and IL-6 (r=-0.170, p=0.032). For the second time point, there was a statistically important positive correlation between TSH and IL-6 (r=0.209, p=0.011) and negative correlation for fT3 and CRP (r=-0.304, p=0.000), fT3 and LEU (r=-0.195, p=0.017), and T4 and IL-6 (r=-0.179, p=0.029). For the third time point, there was a positive correlation between TSH and CRP (r=0.263, p=0.006), TSH and IL-6 (r=0.349, p=0.000), and a negative correlation between TSH and LEU (r=-0.396, p=0.000), fT3 and LEU (r=-0.327, p=0.001), and fT4 and CRP (r=-0.202, p=0.038). Laboratory results are displayed in **Tables 3**–**5**.

We verified if the presence of anti-thyroid antibodies can take its toll on the thyroid function in COVID-19. The presence of anti-TPO abs was confirmed in 13 out of 164 (7.9%) patients and anti-TG abs – in 12 out of 165 (7.3%). TSH levels in patients with positive anti-TG and anti-TPO abs were checked over all three time points. There were considerable differences in TSH levels between the groups with positive and negative autoantibodies, but only for the first time point (p<0.05). At the same time, TSH concentration in patients with anti-TG abs was significantly lower over all three time points (#1: p<0.0001; #2: p<0.005; #3: p<0.005). As we assessed the relationship between the presence of anti-thyroid antibodies and the endpoint completion, we found no significant results.

## Discussion

Most articles on thyroid function in COVID-19 involve HPT parameters assessed over a single time point (15–19). The majority of authors decided to exclude patients treated with GCs as GCs can influence the HPT axis (14, 19, 20). In our project, we decided not only to evaluate hormonal parameters, but to seek for potential trends appearing over the course of prolonged observation (up to 10 days post-admission). We chose to include patients treated with GCs and compare them with their counterparts who did not receive GCs. We evaluated the thyroid function abnormalities and their potential effect on the clinical outcomes.

The available data shows that between 13% and 78% of COVID-19 patients display abnormal thyroid function and the degree of dysfunction differs based on the disease severity (7, 15, 20, 21). This is in accordance with our own research which documented thyroid function pathology, defined as typical laboratory abnormalities within at least one assessed parameter, in more than 80% of all recruited patients over the period of observation. The percentage of affected individuals raised over time, with more than 70% patients with abnormal thyroid function over the third time point of follow-up. These findings support the observations made by Campi and colleagues who decided to record hormonal trends over an extended period (18).

The hormonal pattern we noticed was characteristic for NTI and the results were comparable between the GCG and the NGCG (NTI criteria used: low fT3 and/or low fT4, and/or low T4, and/or elevated rT3, and/or low TSH). However, some of the observed abnormalities, such as decreased TSH and fT3, especially within the GCG, could be explained not only by the NTI, but the use of exogenous GCs as well. The thyroid function abnormalities in NTI, also referred to as euthyroid sick syndrome or low triiodothyronine syndrome, are a complex topic. The pathogenesis might involve excessive generalized immune response and cytokine storm, direct cytotoxic effect of SARS CoV-2 on pituitary and thyroid cells (9–12), altered activity of deiodinases (especially if the concomitant disease is severe), changes in concentrations of carrier proteins for thyroid hormones, changes of thyroid hormone carriers activity (9), endogenous dopamine release, and non-neoplastic hypercortisolemia (18, 20, 22).

Unfavorable clinical prognosis is common in patients with NTI and COVID-19 who did not receive GCs (9, 15, 21, 23). Our project demonstrated that low fT3 can precede unfavorable clinical outcomes both in patients treated and not treated with GCs. As Campi and colleagues offered a longer follow-up, their data revealed that the thyroid profile tends to reverse back to normal in survivors but remains abnormal in those who eventually die (18).

Our analysis proved that HPT dysfunction might predict the occurrence of the combined primary endpoint, and among the secondary endpoints, there is a statistically significant chance for the patients with abnormal thyroid function to require a prolonged hospitalization. A relationship between thyroid function pathology and the clinical course of COVID-19 was confirmed by other authors (14, 16, 23). Zhang and colleagues in their evaluation of COVID-19 patients with abnormal thyroid function described an increased incidence of severe form of the disease, requiring antibiotic treatment, GCs, high-flow oxygenation, non-invasive ventilation, or invasive ventilation. Low triiodothyronine syndrome was the most prevalent clinical entity in the studied group (16).

To fully assess the thyroid function, we evaluated not only the tropic and peripheral hormones, but checked the anti-thyroid antibody titers and the potential relationship between their presence and the disease severity. COVID-19 patients with positive anti-TG abs displayed significantly lower concentration of TSH in all three preset time points as compared with their counterparts with negative antibodies; however, patients with positive autoantibodies did not show a higher likelihood of meeting the endpoints. Lui and colleagues assessed the anti-thyroid antibodies in COVID-19 during hospital stay and three months after the discharge and their follow-up did not confirm a long-standing relationship between the presence of autoantibodies at the baseline and worse clinical outcomes but, quite on the contrary, patients with positive autoantibodies took shorter to recover from symptomatic COVID-19 (23)

Available studies confirmed a link between thyroid function and inflammatory markers, such as ferritin, fibrinogen, erythrocyte sedimentation rate, interleukin-8, interleukin-15, or LYMPH (14, 20, 24). Typically, the more severe the infection was, the more pronounced the abnormalities of thyroid function and the degree of inflammation were. In our material, we noticed multiple various correlations between the results of thyroid function tests and inflammatory markers, with CRP, LEU, and IL-6 being emphasized the most. The variety of observed abnormalities highlights the complex background of the process and most likely arises from different physiopathological processes. We paid special attention to IL-6 known as a potential trigger of cytokine storm, which is often the underlying cause of a severe disease (18, 22, 25, 26) and might influence the thyroid function tests (22). The reverse correlation between the basic thyroid function parameters, such as TSH or fT3, and inflammatory markers was reported before (18), and additional studies confirmed that thyroid dysfunction seemed more pronounced in severe states (19). While low fT3 in most cases can be viewed as a manifestation of NTI, especially with fT4 and TSH decreased and/or within the normal limits, and without clinical signs and symptoms of thyroidopathy, the physiopathological background of decreased TSH may be more complex. We agree with other authors that multiple explanations of this phenomenon in COVID-19 should be considered (18, 22, 27). For instance, Croce et al. in their review of thyroid function in COVID-19 explained that multiple proinflammatory cytokines can influence the HPT, eventually leading to NTI and a drop in peripheral thyroid hormones and/or TSH (22). Other plausible explanations of reduced TSH concentrations include the development of typical or atypical subacute thyroiditis, the inhibitory effect of systemic GCs on the pituitary, or thyrotoxicosis.

As described above, the topic of thyroid function in COVID-19 can be complex. Assessment of the thyroid panel early during hospitalization – as early as on day one – can benefit the patients and allow an early recognition of potential warning signs. Thus, a simple laboratory profile of basic parameters such as TSH, fT3, and fT4 could be implemented into routine diagnostics, with additional clinical value added by repeated testing in different time-points. NTI is common in COVID-19 and, often, the more pronounced it is, the poorer the prognosis is. The abnormalities typical for NTI should reverse once the patient recovers. Nonetheless, some patients might suffer from subacute thyroiditis, hypothyroidism, or thyrotoxicosis. In such cases, clinical signs and symptoms typical for suspected disorders should be assessed together with laboratory (full thyroid profile, anti-thyroid antibodies, sedimentation rate, CRP) and radiological (thyroid ultrasound) work-up, and disease-specific treatment should follow once a diagnosis is concluded.

Strengths of our research are: i. prolonged observation covering three separate time points; ii. inclusion of patients treated with GC who were underrepresented in previous studies; iii. wide range of assessed parameters, including rT3 and IL-6; iv. evaluation of relationship between endpoint completion and thyroid hormone levels; v. unified approach to patient recruitment, blood collection, and follow-up.

However, our study also has its weaknesses: i. recruitment in only one center; ii. ethnically uniform studied group; iii. division of rT3 assessment between two separate laboratories; iv. lack of follow-up focused on detailed clinical signs and symptoms typical for thyroid disorders; v. lack of post-discharge follow-up.

## Summary

Our research proves an important role of endocrine function in COVID-19. Thyroid function markers, especially fT3, are important for the diagnostic process and allow early recognition of unfavorable outcomes. There is a close relationship between thyroid function and the degree of systemic inflammation in COVID-19 patients. Simple hormonal tests like fT3 could predict the hospitalization outcomes, especially in patients with severe disease. Our analysis supplements and confirms the data on observed HPT abnormalities in COVID-19.

## Data Availability Statement

The original contributions presented in the study are included in the article/supplementary material. Further inquiries can be directed to the corresponding author.

## Ethics Statement

The studies involving human participants were reviewed and approved by Independent Bioethics Committee for Scientific Research at the Medical University of Gdańsk. The patients/participants provided their written informed consent to participate in this study. The Independent Bioethics Committee for Scientific Research at the Medical University of Gdańsk waived the requirement of written consent in patients unable to to provide it due to bad general state caused by COVID-19 and/or concomitant diseases.

## Author Contributions

RŚ-S – manuscript concept and preparation, results interpretation, manuscript revision, literature collection and review, project supervision. AB – manuscript concept and preparation, database creation, results interpretation, literature collection and review. ER – patient recruitment, data collection. All authors contributed to the article and approved the submitted version.

## Funding

The study was funded by the National Centre for Research and Development, project number SZPITALE-JEDNOIMIENNE/46/2020.

## Conflict of Interest

The authors declare that the research was conducted in the absence of any commercial or financial relationships that could be construed as a potential conflict of interest.

## Publisher’s Note

All claims expressed in this article are solely those of the authors and do not necessarily represent those of their affiliated organizations, or those of the publisher, the editors and the reviewers. Any product that may be evaluated in this article, or claim that may be made by its manufacturer, is not guaranteed or endorsed by the publisher.
